# Concentration-Dependent Effects of Salicylic Acid and Methyl Salicylate on Chlorophylls, Carotenoids, Fatty Acids, and Phenolic Compounds in the Green Microalga *Planktochlorella nurekis*

**DOI:** 10.3390/md24080283

**Published:** 2026-08-17

**Authors:** Jan Cichoński, Patrycja Michalik, Wiktor Nowak, Paweł Czerniewicz, Ewelina Kuna, Magdalena Słowik-Borowiec, Grzegorz Chrzanowski

**Affiliations:** 1 Doctoral School, University of Rzeszów, 16C Rejtana Street, 35-959 Rzeszów, Poland; janc@dokt.ur.edu.pl; 2 Faculty of Biotechnology, Collegium Medicum, University of Rzeszów, 8B Zelwerowicza Street, 35-601 Rzeszów, Poland; 3 Institute of Biological Sciences, Faculty of Natural Sciences, University of Siedlce, 14 Prusa Street, 08-110 Siedlce, Poland; 4 Faculty of Biology, Nature Protection and Sustainable Development, University of Rzeszów, 4 Zelwerowicza Street, 35-601 Rzeszów, Poland

**Keywords:** *Planktochlorella nurekis*, salicylic acid, methyl salicylate, bioactive compounds, carotenoids, fatty acids, phenolic compounds, L-phenylalanine ammonia-lyase

## Abstract

**Highlights:**

**What are the main findings?**

- Exogenous application of salicylic acid (SA) and methyl salicylate (MS) modified microalgal growth and altered fatty acid profiles.
- The application of 10 µM MS increased pigment levels, and doubled the activity of L-phenylalanine ammonia-lyase (PAL).

**What are the implications of the main findings?**

- Optimized salicylate elicitation can be an effective strategy to enhance the production of valuable compounds, such as monounsaturated fatty acids and carotenoids.
- The upregulation of PAL and the increase in total phenolic content after treatment with 10 µM MS suggest that the phenylpropanoid pathway in *Planktochlorella nurekis* can be induced with MS.

**Abstract:**

Microalgae are a rich source of bioactive metabolites, including unsaturated fatty acids, chlorophylls, carotenoids, and phenolic compounds with antioxidant and nutraceutical potential. Salicylic acid (SA) and its volatile ester, methyl salicylate (MS), are well-known elicitors of secondary metabolism in higher plants, yet their mechanisms of action in green microalgae, particularly the effects of MS, remain poorly understood. Therefore, we evaluated how salicylates applied at concentrations of 0.1, 1, and 10 µM affect the growth and accumulation of bioactive compounds in a high-productivity clone of *Planktochlorella nurekis*, combining gas chromatography coupled mass spectrometry (GC-MS) fatty acid profiling with spectrophotometric determination of pigment levels, total phenols, and L-phenylalanine ammonia-lyase (PAL) activity. SA increased cell number and size, whereas 0.1 µM MS resulted in a six-fold gain in cell number. Accordingly, 10 µM MS raised chlorophyll *b* levels—nearly three-fold (from 76 to 211 µg per g D.W.)—and total carotenoids—roughly four-fold (129 to 520 µg per g D.W.)—while both elicitors significantly increased the monounsaturated fatty acid fraction (from 29% to about 40%). Notably, 10 µM SA lowered the levels of saturated fatty acids. Moreover, 10 µM MS doubled the PAL activity and increased the total phenols by 17% compared to the control. Thus, SA and MS act as effective elicitors of high-value metabolites in *P. nurekis*.

## 1. Introduction

Microalgae are unicellular photosynthetic microorganisms that produce a broad range of biologically active chemicals. Carbohydrates, amino acids, proteins, and lipids are essential for sustaining life, providing cell structure, nutrition, and proliferation, whereas pigments and phenolic compounds serve photoprotective and defense functions and accumulate in response to changing environmental conditions. Because of their role, these compounds exhibit strong antioxidant, antimicrobial, and health-promoting bioactivities; thus, microalgae are considered valuable for food, nutraceutical, and pharmaceutical industries [1].

Microalgae produce a wide array of lipids that can be classified into two main groups: polar, consisting of phospholipids and glycolipids, and nonpolar (neutral) lipids, divided into sterols, triacylglycerols, and fatty acids [2]. Typically, the lipid content in microalgae ranges from 20 to 50% of the dry weight (D.W.) of cells, and under certain conditions, such as nitrogen limitation or intense light, it can increase to 70–80%. Therefore, several strategies, such as regulating nutrient availability and environmental conditions, are employed to produce higher amounts of microalgal lipids, including triacylglycerols (TAGs)—used to obtain biofuels—and unsaturated fatty acids—used in the production of nutraceuticals [3,4]. The biosynthesis of fatty acids occurs in chloroplasts, in which acetyl-CoA is carboxylated to malonyl-CoA, producing saturated fatty acids, which are further desaturated and elongated to form unsaturated fatty acids [5]. Microalgal fatty acids are incorporated into food and nutritional supplements due to their antioxidant, antimicrobial, and anti-inflammatory properties [6]. Supplementation with unsaturated fatty acids may protect from cardiovascular diseases, such as atherosclerosis and atrial fibrillation, and can promote healthy blood lipid profiles and pressure, regulate glucose levels, improve insulin sensitivity, and decrease abdominal obesity [7,8].

Microalgae are a natural source of pigments, such as chlorophyll, which give cells their green color and are essential to photosynthesis. Chlorophylls are oil-soluble, amphiphilic pigments based on a porphyrinic structure consisting of four pyrrole rings and a magnesium ion at the center that play a key role in photosynthesis [9]. The main photosynthetic pigment is chlorophyll *a*, which accounts for about 5% of dry biomass weight in *Chlorella* sp. cells. Within the light-harvesting antennae, chlorophyll *a* absorbs light directly and accepts excitation energy from accessory pigments. Moreover, it acts as a primary electron donor in the migration of this energy to the reaction centers of photosystems I and II. The second most abundant photosynthetic pigment is chlorophyll *b*, an accessory pigment of the light-harvesting apparatus that extends the absorption spectrum and transfers excitation energy to chlorophyll *a*. The ratio of chlorophyll *a* to *b* is usually 3:1 in green algae [10,11]. The production of chlorophyll in microalgae can be affected by several environmental factors, such as light intensity, duration of light/dark cycle, temperature, nutrient availability, and carbon source used by microalgae [12]. The chemical structure of chlorophyll enables its potent biological activity, which can be explored for medical applications. Chlorophyll and its derivatives exhibit antioxidant, antimicrobial and anti-inflammatory activities and in vitro studies suggest their anticancer activity by targeting multiple oncogenic signaling pathways and transcription factors, such as NF-κB [13].

In addition to chlorophylls, microalgae produce carotenoids, which are natural pigments characterized by their yellow, orange, and red colors, from which β-carotene, lutein, violaxanthin, and zeaxanthin were identified as the most abundant within their cells [14]. Furthermore, astaxanthin and other carotenoids do not occur in many higher plants, but they are more commonly synthesized by microalgae [15]. Carotenoids are tetraterpene molecules made of eight isoprene (C5) units that form a forty-carbon skeleton (C40). They are divided into carotenes, which are polyunsaturated hydrocarbons, and xanthophylls—oxidized derivatives of carotene [15,16]. These pigments protect the photosynthetic apparatus against photooxidation from excess absorbed light and stabilize the structural integrity of light-harvesting complexes [17]. Carotenoids in microalgae are formed from the five-carbon isopentenyl pyrophosphate (IPP) and its isomer, dimethylallyl diphosphate (DMAPP) through the plastidial 2-C-methyl-D-erythritol 4-phosphate (MEP) pathway [18]. Carotenoid yield can be increased under nutrient deprivation or environmental stresses, such as high light, temperature, or salinity. Although stress conditions can inhibit growth and induce oxidative damage in cells, many strategies have been developed to overcome these limitations, including two-stage cultivation and the addition of growth-promoting agents [19]. As with chlorophylls, the chemistry of carotenoids, such as functional cyclic end groups, enables their potent antioxidant activity. Furthermore, carotenoids are natural inhibitors of NF-κB signaling, thereby displaying anti-inflammatory properties [20]. Due to the bioactivities of carotenoids, their role in treating various diseases has been thoroughly investigated. They can scavenge free radicals and can protect against diabetes, obesity, age-related macular degeneration, atherosclerosis, non-alcoholic and alcoholic fatty liver, depression, and exhibit anti-aging effects. Additionally, dietary carotenoid intake can reduce the risk of developing various cancers [21].

Another group of metabolites biosynthesized by microalgae is phenolic compounds, a large family of molecules that contain at least one aromatic ring bearing one or more hydroxyl groups. The main identified microalgal groups of phenolic compounds are phenolic acids derived from hydroxybenzoic and hydroxycinnamic acids, and flavonoids [22]. Microalgal phenolics are involved in responses to environmental stresses, such as heavy metals, UV radiation, and high temperatures. Furthermore, they can enhance nutrient uptake through their ability to chelate metal ions. The biosynthesis mechanisms of phenolic compounds in microalgae are not yet fully known. However, in plants, phenolic compounds are derived from the phenylpropanoid biosynthetic pathway, which begins with the formation of trans-cinnamic acid and p-coumaric acid from the amino acids L-phenylalanine and L-tyrosine, catalyzed by L-phenylalanine ammonia-lyase (PAL, EC 4.3.1.24) and L-tyrosine ammonia-lyase (TAL, EC 4.3.1.23), respectively [23,24]. Conditions such as light intensity, UV irradiation, salinity, and nutrient availability strongly affect the composition and content of microalgal phenolic compounds. These metabolites can scavenge ROS, thereby enhancing the antioxidant capacity of cells [25]. They can also chelate transition metals, such as iron and copper, and suppress the Fenton reaction [26]. Furthermore, they possess antimicrobial activity [27] and may protect against diabetes, obesity, and cardiovascular diseases. Moreover, polyphenols may suppress carcinogenesis and tumor formation. Microalgal extracts containing phenolic compounds have also shown anti-inflammatory activity [28].

A number of environmental parameters affect the growth and physiology of microalgae [29], among which temperature is crucial. This factor is a major environmental stressor that causes morphological, physiological, and biochemical changes, and reduces photosynthesis and carbon fixation in cells [30]. On the other hand, different photoperiods may reduce the growth rate and biomass productivity. Continuous illumination stimulated the growth of *Botryococcus braunii* and *Scenedesmus obliquus*, whereas the growth of three microalgal species from the genus *Neochloris* was spurred under a shorter duration of light of the same intensity. Thus, the algae species can be classified into different groups according to their growth ability under different light regimes [31]. Moreover, plant hormones (phytohormones) have also been reported to increase the growth of microalgae by controlling internal biochemical pathways [32,33,34]. Salicylic acid (SA) is one of the most commonly reported plant hormones that modifies growth and the production of secondary metabolites [35]. It activates the plant responses to biotic and abiotic stressors and modulates their growth through crosstalk with other phytohormones, such as auxins and jasmonic acid [36]. It is a vital component of systemic acquired resistance (SAR), in which SA levels in plants increase in response to infection, thereby initiating defense responses and providing protection against a variety of biotrophic pathogens. Furthermore, SA is methylated to produce volatile methyl salicylate (MS), which serves as a long-distance defense signaling molecule and is converted back to SA at its destination. Although methylation reduces the biological activity of SA, it promotes volatility and increases lipophilicity, which may influence the uptake of MS by the cells [37,38]. Whether this relationship is conserved in green microalgae is unknown. Currently, the homologs of nonexpressor of pathogenesis-related genes 1 (NPR1), which mediates SA signaling in plants, have not yet been found in Chlorophyta [39]. Moreover, to our knowledge, SA and MS have not been applied in parallel to microalgal culture, and the reported effects of SA are scarce. Thus, it is not known whether their mechanism of action in microalgae will be similar to that in higher plants, and whether MS will become active in their cells, acting as a defense signal.

We therefore hypothesized that applied SA and MS would spur changes in the growth and secondary metabolism of green microalgae by increasing the accumulation of chlorophylls, carotenoids, phenolic compounds, and fatty acids (particularly unsaturated), highlighting that applied phytohormones could be important elicitors inducing the biosynthesis of bioactive metabolites. The research was conducted on a *Planktochlorella nurekis* clone previously modified by [40], selected for its high productivity of bioactive metabolites, which is attributed to karyokinesis and elevated DNA content. *P. nurekis* cells were treated with three different concentrations (0.1, 1, 10 µM) of SA and MS, selected after Horbowicz et al. [41]. The results of this research may elucidate the roles of SA and MS in the secondary metabolism of green microalgae, providing a more comprehensive understanding of algal biosynthetic pathways and enriching microalgal biotechnology.

## 2. Results

### 2.1. Planktochlorella nurekis Growth

**Figure 1:**
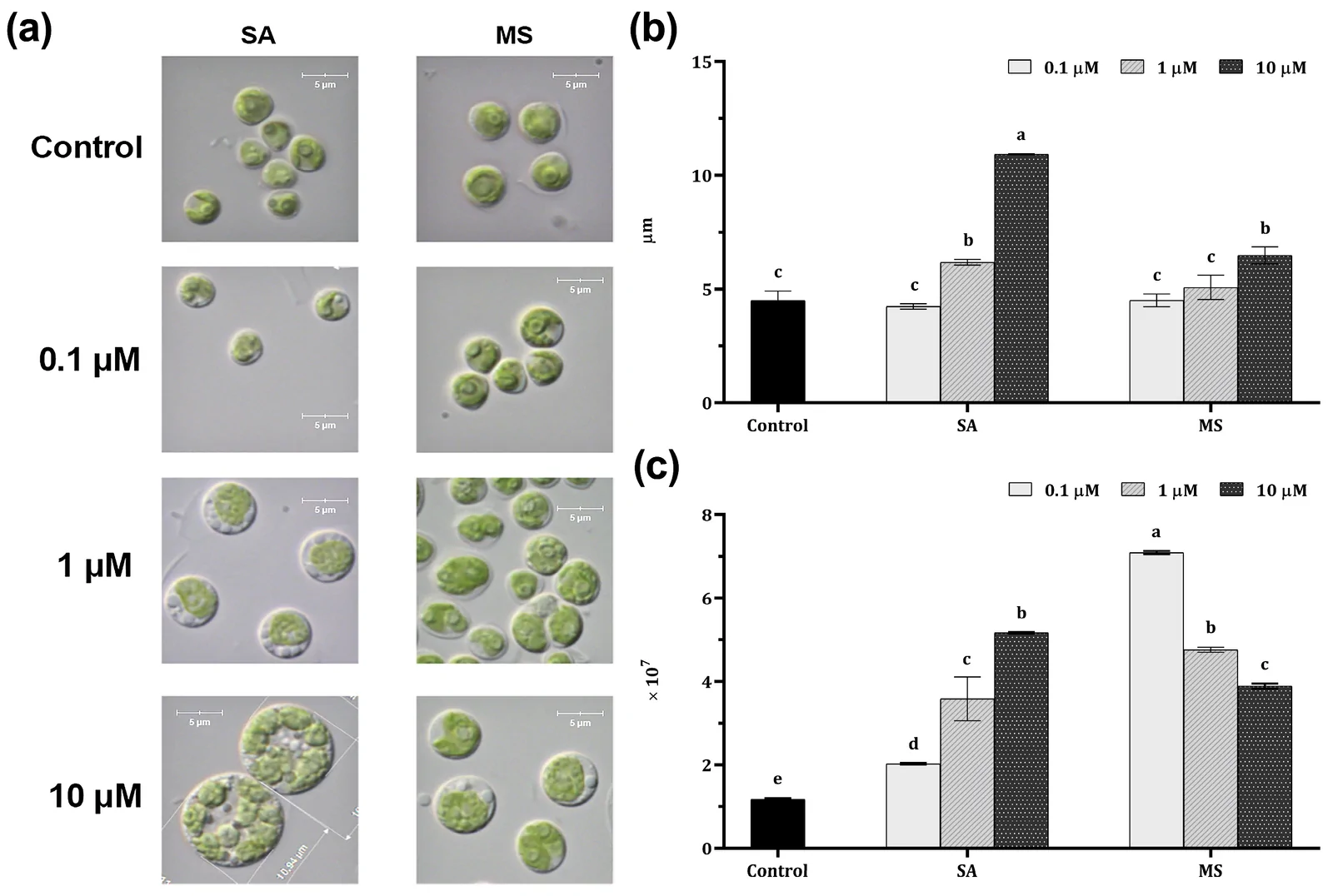
*Planktochlorella nurekis* growth parameters under different concentrations of salicylic acid (SA) and methyl salicylate (MS). (**a**) Photos of *P. nurekis* cell size. (**b**) Cell size measurements. (**c**) Changes in cell number per mL. Columns (separately in (**b**,**c**)) marked by different letters are statistically different at *p* ≤ 0.05 (Tukey’s test).

Following exposure to salicylates, *Planktochlorella nurekis* cells showed significant changes in their size and number. The application of 0.1 µM salicylic acid (SA), as well as two lower concentrations (0.1, 1 µM) of methyl salicylate (MS), did not result in any significant change in *P. nurekis* cell size (Figure 1a,b), while the treatment with 1 µM SA and 10 µM MS caused a slight but significant increase in cell size. However, the highest and most significant increase in cell size was observed in microalgae treated with 10 µM SA. Moreover, the treatment with SA resulted in a gradual increase in cell number (Figure 1c), reaching 5.17 × 10^7^ × mL^−1^ (compared to 1.17 × 10^7^ × mL^−1^ in the control). Conversely, the number of *P. nurekis* cells gradually decreased with rising concentrations of MS. Nevertheless, the application of 0.1 µM MS resulted in the highest six-fold increase in the number of cells in microalgae cultures compared to the control (from 1.17 × 10^7^ × mL^−1^ to 7.09 × 10^7^ × mL^−1^ cells).

### 2.2. Fatty Acid Profile

**Figure 2:**
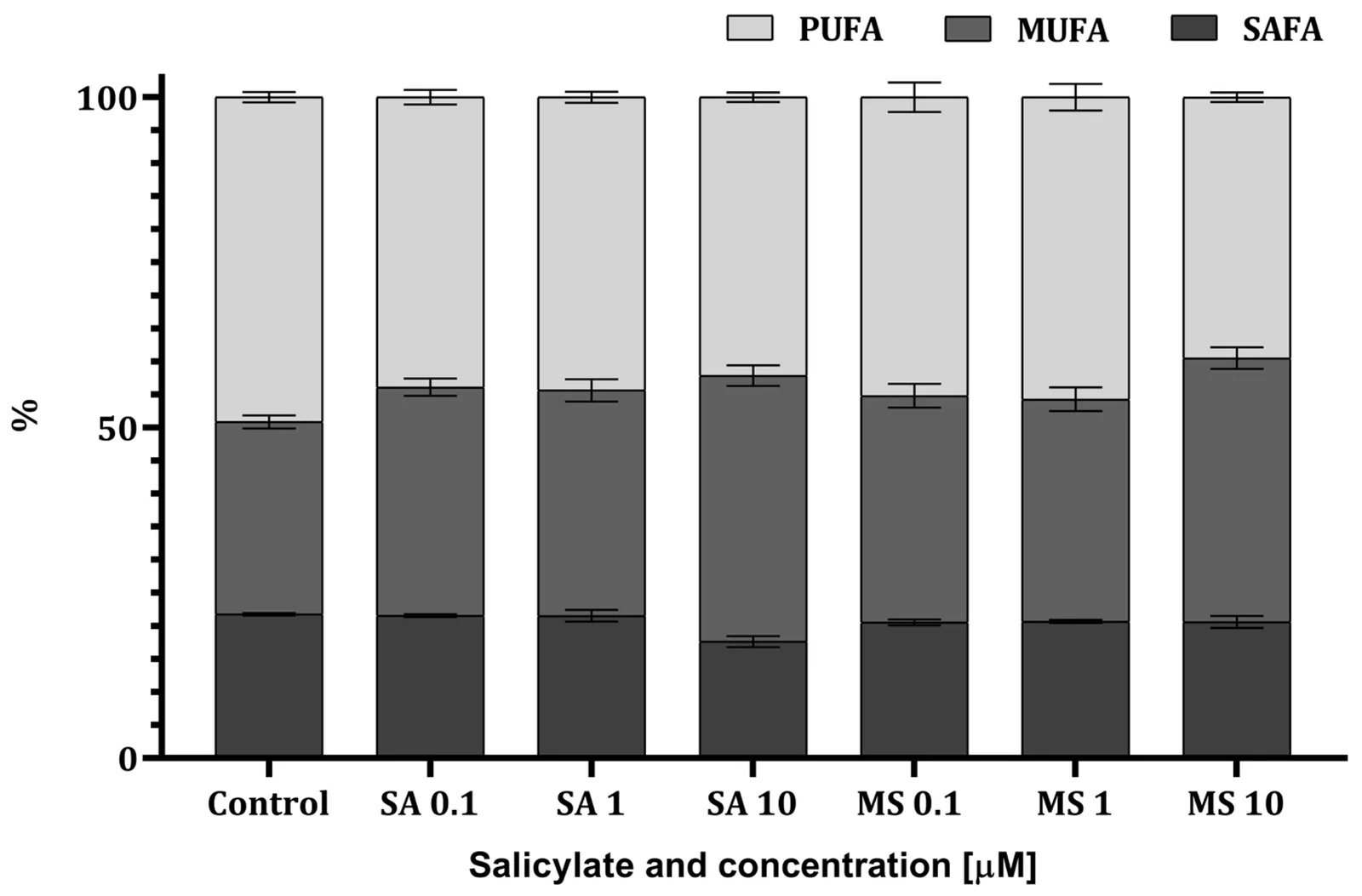
Content of fatty acid fractions (SAFA, MUFA, PUFA) in *P. nurekis* cells.

In the cells of *Planktochlorella nurekis*, 22 fatty acids were identified. Among the three identified groups of compounds, polyunsaturated fatty acids (PUFAs) were the most abundant, constituting approximately half of all detected fatty acids (Figure 2, Table 1). The treatment with 10 µM SA resulted in a significant decrease in the content of the saturated fatty acid fraction (SAFA)—from 21.75 ± 0.19% to 17.64 ± 0.84%. Furthermore, the content of individual SAFAs, such as myristic (C14:0), palmitic (C16:0), margaric (C17:0), stearic (C18:0), behenic (C22:0), tricosylic (C23:0), and lignoceric (C24:0), was also significantly decreased, except for the arachidic acid (C20:0) content, which did not change. However, treatment with 1 µM SA resulted in an increase in the content of arachidic and stearic acids and caused a two-fold increase in the content of margaric acid. On the other hand, treatment with SA and MS caused an increase in the monounsaturated fatty acid fraction (MUFA).

The highest increase in MUFA content was observed in *P. nurekis* cells treated with 10 µM SA (40.22 ± 1.54%) and 10 µM MS (39.92 ± 1.64%), compared to the control (29.11 ± 0.99%). Salicylate treatment did not significantly change the levels of pentadecanoic (C15:1) and nervonic (C24:1n−9) fatty acids. The content of palmitoleic acid (C16:1n−7) increased after treatment with 0.1 µM SA. A two-fold increase in heptadecenoic acid (C17:1) content was observed within the cells treated with all concentrations of SA. Moreover, salicylate treatment (except for 0.1 µM SA) significantly increased the levels of oleic acid (C18:1n−9, cis) in microalgal cells from 24.79 ± 1.08% in the control to 35.94 ± 1.58% in cells treated with 10 µM SA and to 35.99 ± 1.9% in *P. nurekis* cells treated with 10 µM MS; oleic acid represented the highest content of any detected individual fatty acid in the tested *P. nurekis* microalgae. In contrast, the content of the polyunsaturated fatty acid (PUFA) fraction decreased after salicylate treatment. However, the application of 10 µM MS resulted in a two-fold increase in the content of docosahexaenoic (C22:6n−3) and γ-linolenic (C18:3n−6) fatty acids.

### 2.3. Chlorophylls and Carotenoids

**Figure 3:**
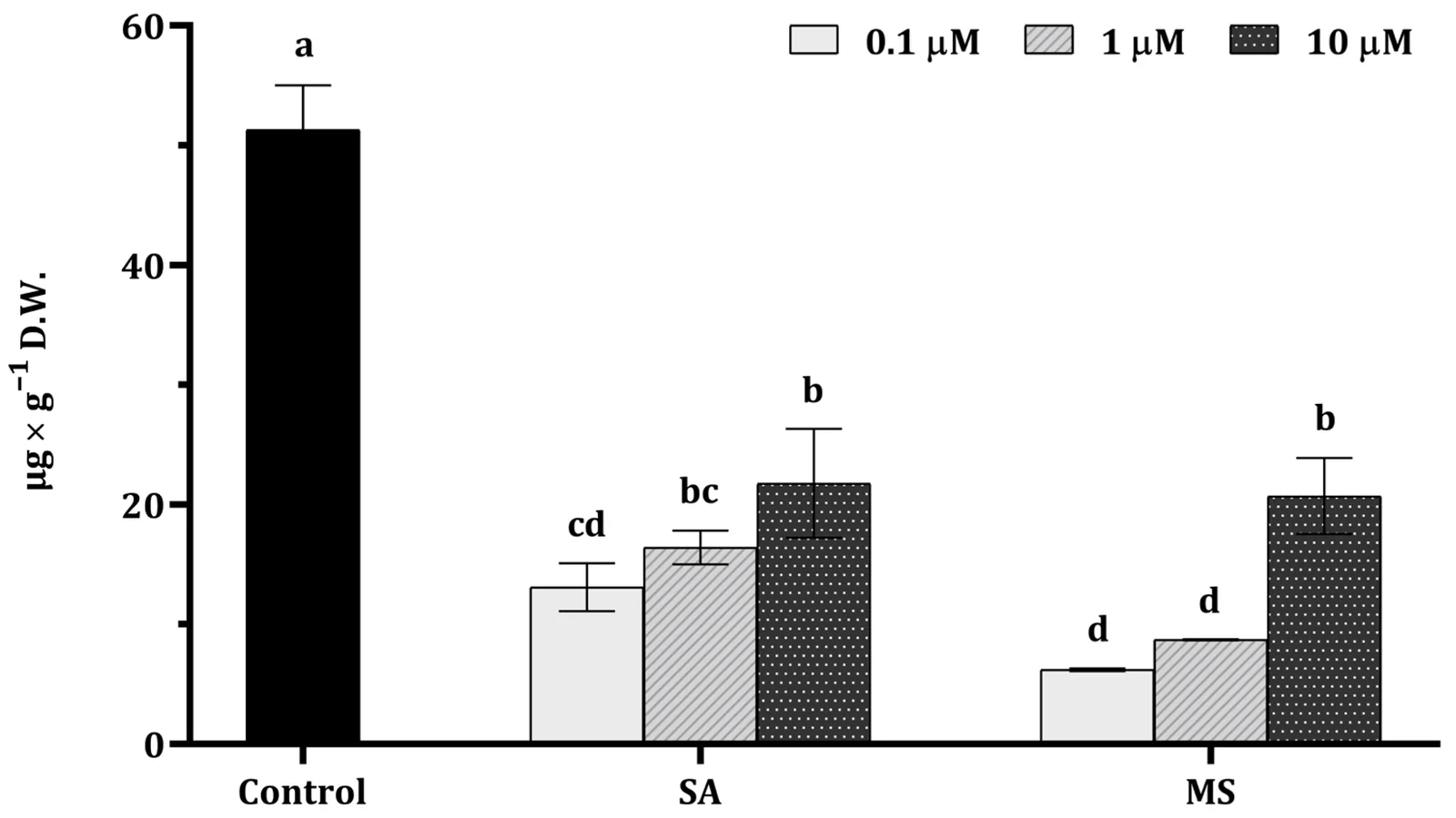
The content of chlorophyll *a* within *P. nurekis* cells treated with different concentrations of salicylic acid (SA) and methyl salicylate (MS), expressed as micrograms per gram of dry weight (µg × g^−1^ D.W.). Columns marked by different letters are statistically different at *p* ≤ 0.05 (Tukey’s test).

The treatment with salicylates caused different effects on the levels of chlorophyll *a* and chlorophyll *b* in *P. nurekis* cells. Generally, a significant decrease in chlorophyll *a* levels (Figure 3) was observed in all salicylate-treated cultures. The smallest decrease in chlorophyll *a* levels relative to the control was reported within microalgal cultures treated with 10 μM SA (21.77 ± 4.55 μg/g D.W.) and 10 μM MS (20.70 ± 3.18 μg/g D.W.), and the difference between these two results was not statistically significant. The lowest total chlorophyll *a* levels were recorded in cultures treated with 0.1 μM MS (6.20 ± 0.10 μg/g D.W) and 1 μM MS (8.65 ± 0.05 μg/g D.W).

**Figure 4:**
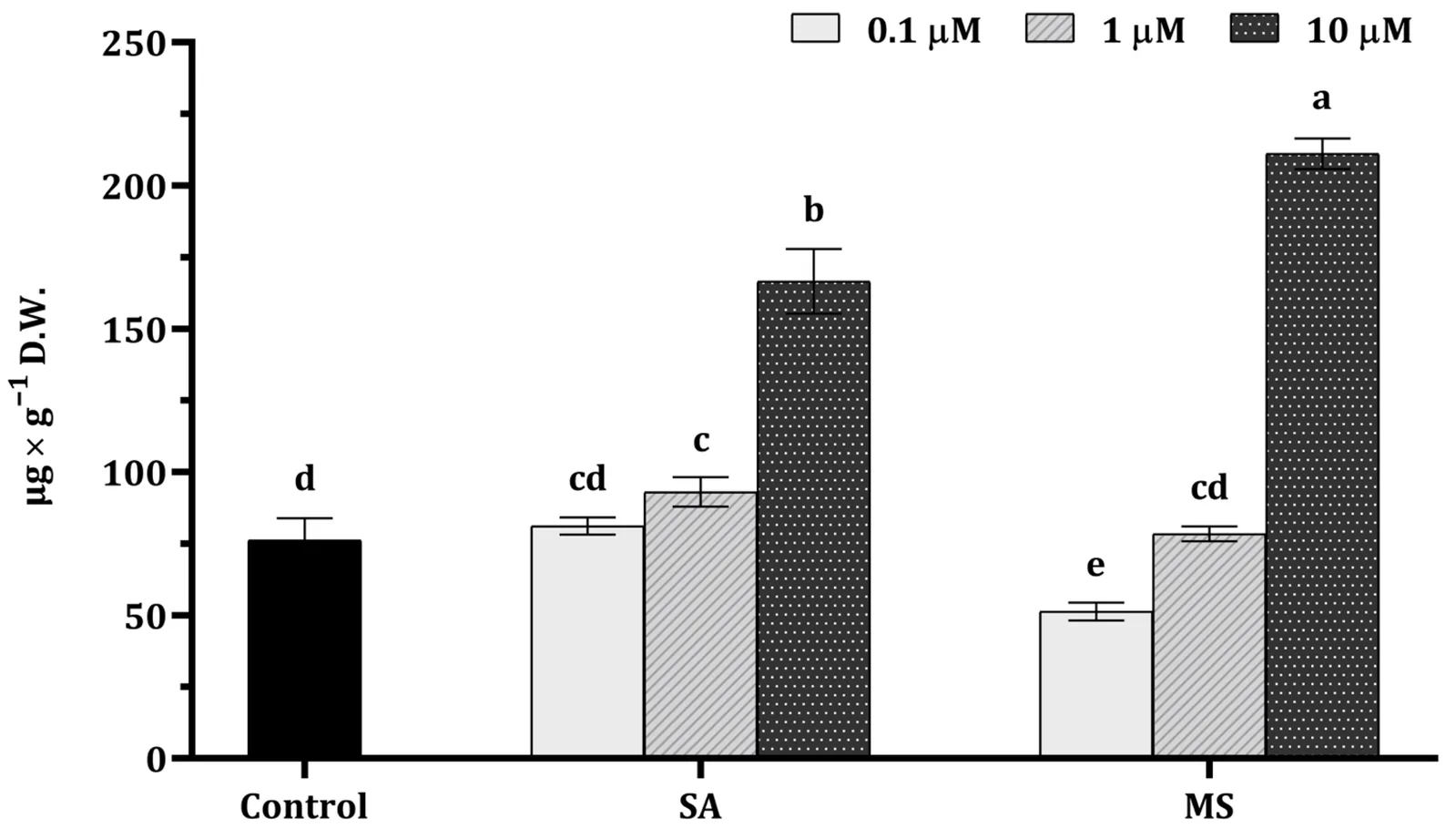
The content of chlorophyll *b* within *P. nurekis* cells treated with different concentrations of salicylic acid (SA) and methyl salicylate (MS), expressed as micrograms per gram of dry weight. Columns marked by different letters are statistically different at *p* ≤ 0.05 (Tukey’s test).

On the other hand, the quantities of chlorophyll *b* (Figure 4) increased after the addition of the highest concentrations of SA and MS to the microalgal cultures, compared to the untreated *P. nurekis* (76.13 ± 7.70 μg/g D.W.). The treatment with 10 μM SA resulted in an increase in chlorophyll *b* levels of 118.8% (166.58 ± 11.32 μg/g D.W.) compared to the control. Furthermore, cells treated with 10 μM MS exhibited the highest chlorophyll *b* levels, reaching 211.10 ± 5.35 μg/g D.W.

**Figure 5:**
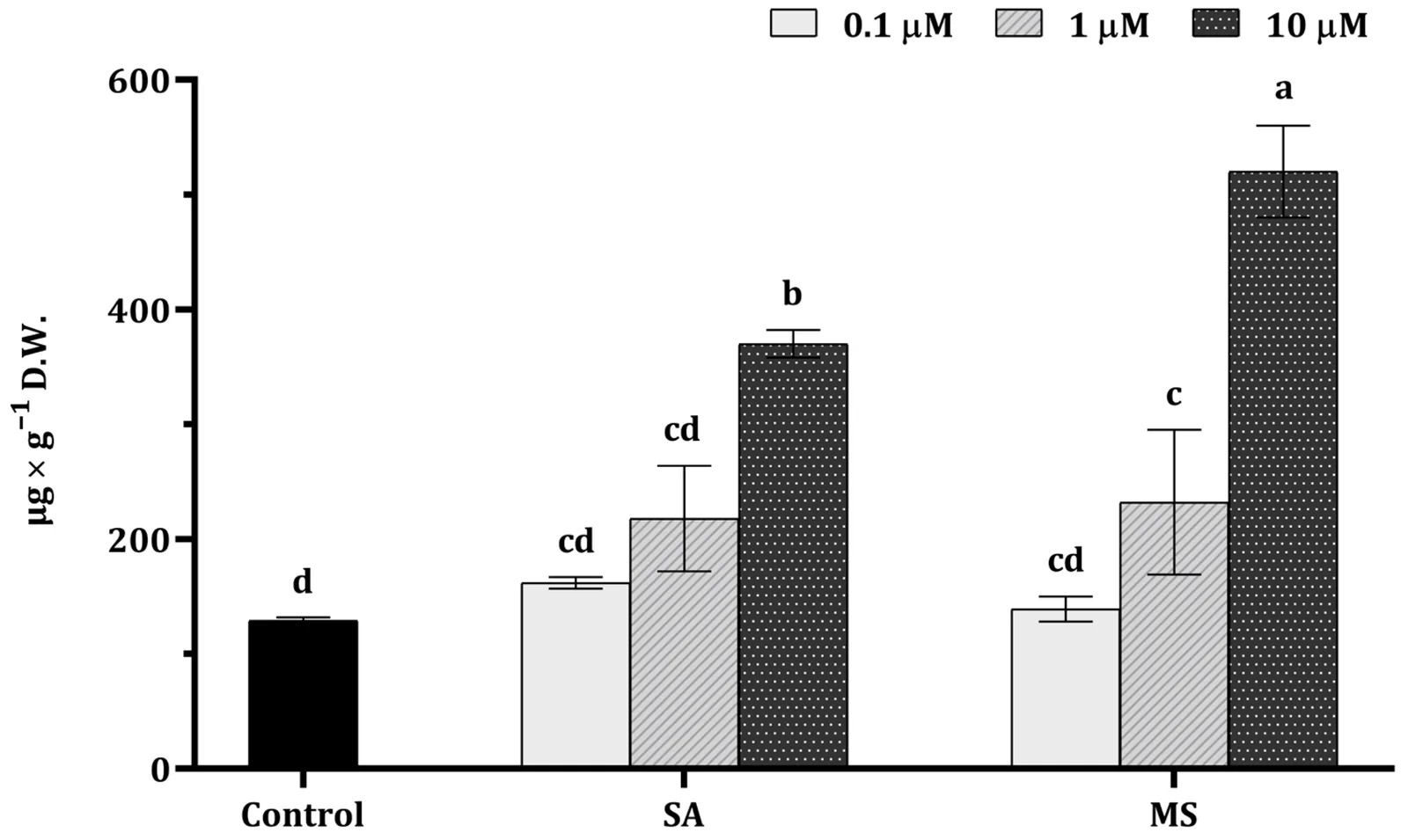
The content of carotenoids within *P. nurekis* cells treated with different concentrations of salicylic acid (SA) and methyl salicylate (MS), expressed as micrograms per gram of dry weight. Columns marked by different letters are statistically different at *p* ≤ 0.05 (Tukey’s test).

The concentration of carotenoids in untreated *P. nurekis* cells was 129 µg per gram of dry weight (D.W). The application of 10 µM MS induced the highest increase in carotenoid levels within the cells—up to 520 µg per g D.W.—compared to the control (Figure 5). Treatments with 0.1 µM and 1 µM SA and the lowest concentration of MS (0.1 µM) caused no significant increase in the total carotenoids within *P. nurekis* cells. The application of 1 µM MS caused an increase from 129 to 232 µg per g D.W. in the content of carotenoids. The 10 µM SA treatment resulted in an increase to 370 µg per g D.W. in total carotenoids compared to the control.

### 2.4. Total Phenols

**Figure 6:**
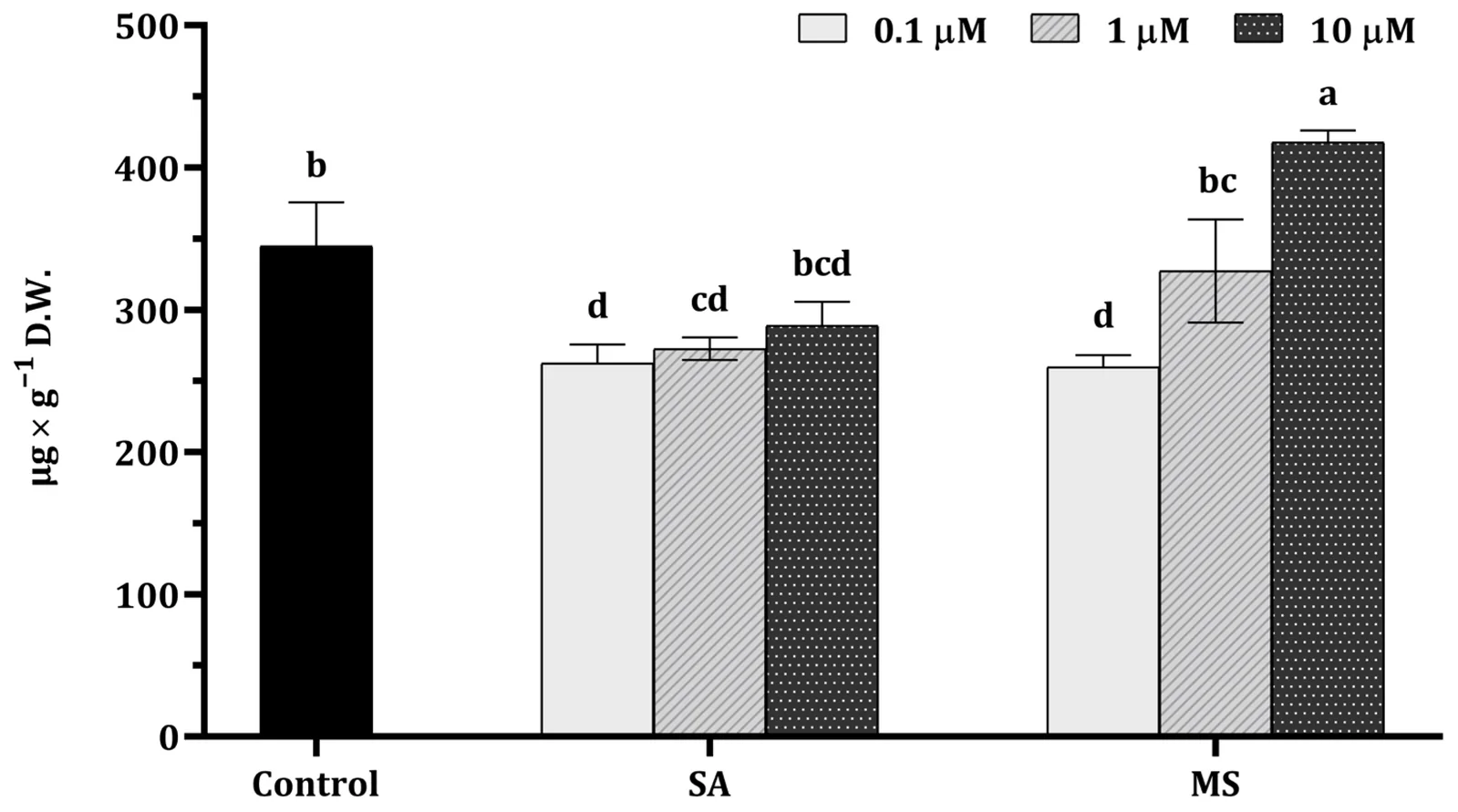
The content of phenolic compounds within *P. nurekis* cells treated with different concentrations of salicylic acid (SA) and methyl salicylate (MS), expressed as micrograms per gram of dry weight. Columns marked by different letters are statistically different at *p* ≤ 0.05 (Tukey’s test).

The highest content of total phenols was noted in *P. nurekis* cells treated with the highest concentration (10 µM) of MS, resulting in an increase of 16.7% (from 345 to 418 µg/g D.W.) compared to the control (Figure 6). The SA treatment showed lower levels of total phenols (262, 273, 289 µg/g D.W.) compared to the control, with no significant increase in total phenols along with rising SA concentrations (0.1, 1, 10 µM). The MS treatment also did not result in higher levels of total phenols compared to the control, except for 10 µM MS. The content of phenolic compounds increased proportionally (260, 327, and 418 µg/g D.W.) to the concentrations of MS (0.1, 1, and 10 µM, respectively), but only microalgae treated with 10 µM MS had higher levels of total phenols within cells compared to untreated *P. nurekis* cells.

### 2.5. L-Phenylalanine Ammonia-Lyase Activity

**Figure 7:**
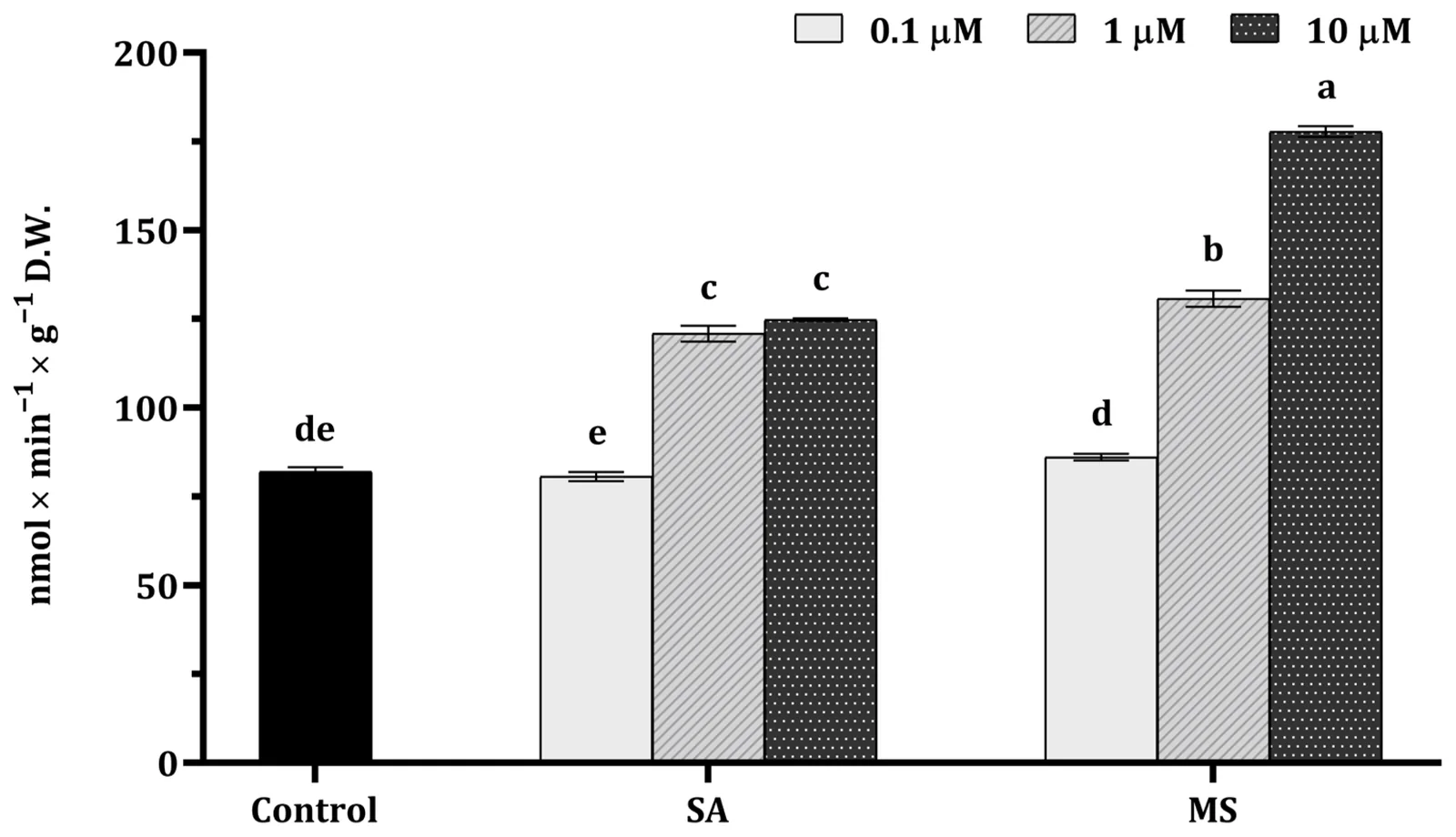
The activity of L-phenylalanine ammonia-lyase (PAL) within *P. nurekis* cells treated with different concentrations of salicylic acid (SA) and methyl salicylate (MS), expressed as nanomoles of trans-cinnamic acid produced in 1 min by 1 g of dry algal biomass. Columns marked by different letters are statistically different at *p* ≤ 0.05 (Tukey’s test).

The analysis of L-phenylalanine ammonia-lyase (PAL, EC 4.3.1.24) activity showed the highest increase relative to the control after the application of the highest concentration of MS (10 µM) (Figure 7). In particular, the application of 10 µM MS resulted in a more than twofold increase in PAL activity (from 81.97 to 177.78 nmol × min^−1^ × g^−1^ D.W.) compared to the control. On the other hand, no significant effect on PAL activity was demonstrated at the lowest concentration of both salicylates (0.1 µM). Treatment of *P. nurekis* with 1 µM SA resulted in a 47.4% increase in PAL activity (to 120.86 nmol × min^−1^ × g^−1^ D.W.) compared to the control. However, using a higher SA concentration (10 µM) did not result in any further increase in PAL activity. A different result was observed after the treatment of *P. nurekis* with MS, in which PAL activity increased proportionally to the concentration of MS, except for the lowest concentration used (0.1 µM). Treatment with 1 µM MS increased PAL activity by 59.5% (from 81.97 to 130.74 nmol × min^−1^ × g^−1^ D.W.).

## 3. Discussion

In the present work, we demonstrated that salicylates elicited concentration-dependent responses in *Planktochlorella nurekis*. Treatment with salicylic acid (SA) and methyl salicylate (MS) resulted in improved cell growth and lipid composition, increased chlorophyll *b* and carotenoid levels, elevated PAL activity, and 10 μM MS promoted phenolic compound accumulation.

The growth-promoting effect of SA observed in *P. nurekis* is consistent with earlier reports. Czerpak et al. [42] showed that SA stimulates algal growth and increases the cell number of *Chlorella vulgaris* with exogenous 10–100 μM SA. The most significant effect on cell number was induced by 100 μM SA, which stimulated cell proliferation by 123–140% compared to the control. Furthermore, Zhang et al. [43] demonstrated that exposure to SA (50, 100, and 500 μM) proportionally increased *Chromochloris zofingiensis* cell number. In contrast, the highest increase in dry weight (D.W.) was observed in cells treated with 50 μM SA, and it decreased with the rise in SA concentration. In higher plants, SA and its methylated ester, MS, can modulate growth by affecting cell division and expansion, with divergent effects depending on concentration, plant species, and target organs [44]. Generally, lower exogenous SA concentrations promote growth, whereas higher concentrations can inhibit plant development. SA signaling is canonically mediated through the NPR1 (nonexpressor of pathogenesis-related gene 1) receptor, a key component of the process of plant immune responses, which binds to SA and initiates changes in the expression of key genes involved in plant growth and development. It was demonstrated that the NPR1 cascade can reprogram up to 20% of the transcriptome in *Arabidopsis thaliana*. Additionally, plant growth and development can be modified by other SA receptors, such as SABPs (salicylic acid-binding proteins), including SABP1 (catalase). Moreover, interactions between SA and other hormones, such as gibberellic acid, can enhance SA biosynthesis and, in effect, improve plant growth [44,45]. However, there is no evidence of NPR occurrence in green microalgae. For instance, Feng et al. [39] did not find homologs of the NPR gene family in the studied Streptophyta and Chlorophyta algae. On the other hand, Fu et al. [46] found that salicylic and benzoic acids upregulate the expression of genes involved in glycolysis, DNA replication, the TCA cycle, and the MAPK pathway in *Chlorella regularis*, thereby regulating its growth and metabolism, in a manner distinct from that in plants. Overall, the changes in microalgal growth induced by SA and MS are significant, and further research should be conducted to investigate the mechanism of SA action on microalgal growth, including subsequent transcriptomic changes and the crosstalk between SA and phytohormones.

The induction of lipid accumulation in microalgae is often achieved through abiotic stress. However, this approach usually leads to oxidative stress, which results in a relatively low growth rate. Salicylates improved the fatty acid profile in *P. nurekis* by increasing the proportion of the monounsaturated fatty acid fraction and decreasing the content of polyunsaturated and saturated forms. Zhang et al. [43] similarly indicated that MUFA levels significantly increased following SA treatment in the green microalga *Chromochloris zofingiensis*, and the levels of SAFA and PUFA decreased. Furthermore, the content of oleic acid (C18:1) was also elevated, which is analogous to the increase in oleic acid content in *P. nurekis* cells after the application of SA and MS. They discovered that the enzymes involved in the degradation pathways of tryptophan, tyrosine, leucine, isoleucine, and lysine, which break down to form acetyl-CoA, were upregulated. Moreover, they found that the expression of pyruvate dehydrogenase, which catalyzes the conversion of pyruvate into acetyl-CoA, and the activity of two key enzymes of fatty acid synthesis: acetyl-CoA carboxylase (ACCase) and 3-Oxoacyl-acyl carrier protein reductase (fabG), were also heightened. Additionally, Xu et al. [47] reported that SA significantly increased fatty acid accumulation in the marine diatom *Phaeodactylum tricornutum* and similarly upregulated fatty acid synthesis genes, such as enoyl-[acyl-carrier-protein] reductase (FabI), 3-oxoacyl-[acyl-carrier-protein] synthase (FabFa), and short-chain dehydrogenase/reductase (SCD), while downregulating two β-oxidation genes (KCT3, ACL1) and decreasing oxidative stress. Moreover, Δ^9^-desaturase (PTD9), which is responsible for catalyzing MUFA formation from their saturated forms, was also upregulated. Banas et al. [48] found that membranes prepared from plants treated with salicylic acid had depressed Δ^12^ fatty acid desaturase activity and decreased PUFA (C18:3) levels, with an increase in MUFA (C18:1). Therefore, the response observed in *P. nurekis* can be associated with increased acetyl-CoA production due to increased amino acid breakdown, heightened activity of pyruvate dehydrogenase and enzymes responsible for fatty acid synthesis and reduced fatty acid degradation. Subsequently, the observed drop in SAFA and increase in MUFA levels may be a consequence of increased PTD9 activity and possibly the decreased activity of Δ^12^ desaturase.

It is well known that microalgae are rich sources of pigments, such as chlorophylls and carotenoids. In our study, the treatment with salicylates, especially at the highest concentrations, increased chlorophyll *b* levels. Mirshekari et al. [49] observed that SA induced chlorophyll *b* and total chlorophyll accumulation in *Dunaliella salina*, which is consistent with our research. However, SA also increased chlorophyll *a* levels, contrasting our results. Furthermore, Czerpak et al. [42] demonstrated that SA in the concentration of 1–100 μM stimulated the accumulation of both chlorophyll *a* and *b*. Various abiotic stressors, such as intense light, high temperature, and nutrient deficiency, can hinder the biosynthesis of chlorophyll and promote its degradation in higher plants [50] and in microalgae [51,52]. However, studies suggest that SA and different phytohormones can increase the accumulation of chlorophyll and other secondary metabolites, potentially reducing the adverse effects of abiotic stress. For instance, Barwal et al. [53] demonstrated that the levels of chlorophyll *a*, *b*, and total chlorophyll decreased under salinity stress in maize. This effect was mitigated by the exogenous addition of SA, which improved salt stress tolerance. Similar results were shown in radish, in which 25–100 mM SA increased the levels of chlorophylls (*a*, *b*, *a* + *b*) in plants cultivated under optimal conditions and subjected to salinity stress [54]. Moreover, SA improved chlorophyll fluorescence in *Scenedesmus obliquus* and *Chlorella pyrenoidosa* exposed to cadmium stress [55]. In contrast, in the green microalga *Scenedesmus quadricauda*, the application of 25 μM SA increased chlorophyll *b* and total chlorophyll levels, but it did not prevent the copper-induced decrease in chlorophyll *a* and total chlorophyll levels, which were lower in the combined SA + Cu variant compared to the Cu treatment alone [56].

Salicylates further promoted the production of carotenoids in a concentration-dependent manner, particularly at the highest concentrations of SA and MS. In line with our results, Czerpak et al. [42] reported an increase of 133–157% in carotenoid accumulation within the *Chlorella vulgaris* microalgae between days 4 and 8 of cultivation after treating the cells with 100 μM SA, suggesting that this compound may act as an elicitor in green microalgae species to promote carotenoid biosynthesis. On the contrary, Du et al. [57] observed that the content of total carotenoids within *Botryococcus braunii* cells declined after treatment with SA but was positively impacted by other elicitors, including 6-BA (6-benzylaminopurine), EBR (epibrassinolide), and particularly NAA (1-naphthaleneacetic acid), which significantly enhanced the carotenoid accumulation within these microalgae. Furthermore, Raman and Ravi [58] found that SA may reduce the level of carotenoids in *Haematococcus pluvialis*, irrespective of illumination intensity. However, astaxanthin levels under low and high illumination conditions increased significantly. This variance suggests that salicylates may modulate pigment biosynthesis pathways differently, depending on species-specific regulatory mechanisms. The mechanism of astaxanthin accumulation in *H. pluvialis* after SA treatment was further explored by Gao et al. [59]. The results suggested that astaxanthin accumulation was increased by the upregulation of eight carotenoid biosynthesis genes at the transcriptional level (such as phytoene synthase—psy), the post-transcriptional level (lycopene β-cyclase gene—*lyc*), and the *pds* gene (phytoene desaturase), which upregulated astaxanthin accumulation at both levels. Similarly, SA increased the activity of key enzymes phytoene synthase (PSY), phytoene desaturase (PDS), and lycopene β-cyclase (LCYB) in *Dunaliella salina* exposed to salt stress, which caused a significant increase in 9-cis-β-carotene, which is a more active isomer of β-carotene but is harder to obtain [60]. The direct influence of salicylates on carotenogenesis may be hinted at by the analysis of the promoters of the *psy* genes encoding phytoene synthase (PSY, EC 2.5.1.32), which catalyzes the first step of the carotenoid biosynthesis pathway. Wang et al. [61] identified several cis components within the promoters of the *psy* gene in tobacco (*Nicotiana tabacum*) that are involved in responses to light, temperature, drought stress, and phytohormones. Among them, they identified the TCA element, which was present only in one of the three tobacco PSY genes (NtPSY2) and plays a role in the response to SA. They concluded that the expression of *psy* genes is regulated by a wide range of developmental and environmental factors through different mechanisms. Thus, SA can elicit the production of desirable carotenoids in microalgae by modulating the activity of key genes and enzymes in the carotenoid biosynthetic pathway. In summary, our results indicate a reorganization of the photosynthetic apparatus, with a decrease in chlorophyll *a* and accompanied by increases in chlorophyll *b* and carotenoids. The decrease in the chlorophyll *a*/*b* ratio suggests a reduction in the size of LHCs, which require chlorophyll b for their assembly, relative to the reaction centers [62,63]. Part of the carotenoid increase may also be associated with this change, since the LHC binds carotenoids alongside chlorophylls and contributes to photoprotection and ROS scavenging [63]. On the other hand, a high chlorophyll *a*/*b* ratio indicates greater acclimation to high light intensities and enhanced photosynthetic electron transport, whereas a lower index indicates shade tolerance [64].

Phenolic compounds are antioxidant secondary metabolites biosynthesized by higher plants and microalgae that protect them against biotic and abiotic stress [23]. The application of SA and the lowest concentration of MS resulted in a decrease in total phenols in *P. nurekis*, and only 10 μM MS induced the accumulation of phenolic compounds in this microalga. Similarly, Tunca et al. [65] demonstrated that the content of total phenols decreased with the rising concentrations of SA in the alga *Arthrospira platensis*. However, the application of SA (in particular 1.5 mM) has increased the levels of total phenols in chickpea [66]. Furthermore, Linić et al. [67] showed that foliar application of salicylic and ferulic acids on Chinese cabbage increased levels of phenolic compounds. It is suggested that the enzymes responsible for the production of key intermediates in the phenolic compound biosynthesis pathway are conserved in all major algal divisions, but the occurrence of PAL, the first enzyme of the phenylpropanoid pathway, within algal taxa is not yet well documented [24]. Additionally, treatment with 1 and 10 μM SA and MS increased the activity of PAL, the first enzyme of the biosynthesis of phenolic compounds, in our *P. nurekis* cells, with the highest increase observed in cells treated with 10 μM MS. This finding corresponds with other research suggesting that salicylates can enhance PAL activity in different plants and further promote phenolic compound accumulation. Gacnik et al. [68] demonstrated that specific PAL activity within the apple fruit peel treated with MS was characterized by an increase in PAL activity by 15% after 6 h and by 55% after MS treatment. In contrast, PAL activity in fruits treated with SA did not differ significantly from the control after 6 h of application, and after 24 h, enzyme activity was slightly increased but remained lower compared to the start of the experiment. The levels of two other key enzymes of the phenolic compounds pathway, chalcone synthase (CHS) and flavanone 3β-hydroxylase (FHT), also increased significantly following MS treatment. Additionally, FHT was upregulated after treating fruits with SA. The increased activity of PAL under MS treatment may be associated with the physical characteristics of MS. Although methylation inactivates SA, it increases its membrane permeability and volatility, thus allowing more effective transport of this defensive molecule. After reaching its target, MS is converted back to SA by the methyl esterase activity of SABP2 (salicylic acid-binding protein 2; EC. 3.1.1.-), to induce SAR defensive response [69]. In summary, the results indicate that salicylates act on *P. nurekis* as elicitors that direct the metabolism towards antioxidant defense. The elevation of PAL activity under both compounds demonstrates that the phenylpropanoid pathway in this microalga is inducible by salicylates. However, the increase in PAL activity was not followed by a rise in the accumulation of phenolic compounds. We suggest that the applied salicylates induced stress conditions, in which these compounds were used in antioxidative defense. Particularly after the application of 10 μM MS in *Planktochlorella nurekis,* a shift from growth towards the production of defensive compounds, such as MUFA, carotenoids, and phenolic compounds, was observed. Furthermore, Czerniewicz et al. [26] suggest a time shift between the enzyme’s activity and the phenylpropanoid or flavonoid compound accumulation under stress conditions. Thus, a similar effect could be present in our microalgae treated with salicylates.

The findings from this research can be useful for microalgal biotechnology and the industrial production of valuable compounds. Microalgae are used commercially due to their ability to accumulate valuable products, such as fatty acids, carotenoids, and other antioxidants, such as phenolic compounds [70]. Conventionally, the induction of high-value metabolites in microalgae requires the implementation of controlled stressors, such as high temperature and intensive light, or the use of genetically engineered strains. However, applying severe stress can be a major bottleneck for the commercialization of useful bioproducts, as it can lead to their heightened accumulation at the expense of growth, resulting in decreased biomass productivity. Moreover, the genetic tools used for microalgae programming need to be improved, and they may pose risks to ecosystems in case of contamination of the local environment by genetically modified microalgae [71,72]. The application of elicitors such as SA and MS offers a scalable and cost-effective alternative. Our results suggest that optimizing the concentration of salicylates can induce the production of compounds related to stress defense, such as monounsaturated fatty acids, chlorophyll *b*, carotenoids, and phenolic compounds. Optimizing salicylate doses can mitigate growth inhibition while maintaining the production of valuable compounds. It is also worth noting that we used lower concentrations of elicitors compared to the discussed literature, which makes this method cost-effective. Moreover, it has been demonstrated that microalgae from the *Parachlorella* clade, which includes *P. nurekis*, can maintain growth and photosynthetic efficiency under high-salt conditions, which can enable large-scale cultivation in environments with reduced availability of fresh water [73,74].

## 4. Materials and Methods

### 4.1. Planktochlorella nurekis Growth

The microalgal samples were obtained from the *Planktochlorella nurekis* strain (BO3), which was previously modified by Szpyrka et al. [40] with cytochalasin B and colchicine to achieve a high productivity of bioactive metabolites. Microalgae were cultivated in glass photobioreactors in a liquid medium containing the following macro- and microelements under continuous artificial LED red light (645 nm) and blue light (460 nm) (ratio 3:1) at 16,000 LUX and 28 ± 1 °C, according to [40]: KNO_3_ (5 g/L), MgSO_4_ (2.5 g/L), KH_2_PO_4_ (1.25 g/L), (NH_4_)_2_SO_4_ (0.6 g/L), FeSO_4_ × 7H_2_O (3.7 mg/L), MnCl_2_ × 4H_2_O (2.2 mg/L), H_3_BO_3_ (1.5 mg/L), CoCl_2_ × 6H_2_O (15 µg/L), ZnSO_4_ × 7H_2_O (0.25 mg/L), CuSO_4_ × 5H_2_O (0.25 mg/L), NiSO_4_ × 6H_2_O (0.5 mg/L), NH_4_VO_3_ (23 µg/L), MoO_3_ (18 µg/L), Na_2_WO_4_ × 2H_2_O (0.16 mg/L), KI (0.05 mg/L), and EDTA (0.04 g/L) (Chempur, Piekary Slaskie, Poland). The initial cell density was 1 × 10^6^ cells × mL^−1^. Microalgal cultures were treated with salicylic acid (SA) and methyl salicylate (MS)—applied once to the microalgal growth medium at concentrations of 0.1, 1, and 10 μM after the doubling of cell number. Then, the microalgae were further cultivated for 7 days. The size of microalgal cells was analyzed using an Olympus BX61 differential interference contrast microscope—equipped with a DP72 CCD camera—and the Olympus CellF software (Olympus, Warsaw, Poland). The cell number was determined by directly counting the cells in the growth medium using a TC10 automated cell counter (Bio-Rad, Warsaw, Poland). Prior to the analysis, the biomass was harvested by centrifugation and freeze-dried.

### 4.2. Fatty Acid Profile

Briefly, 10 mg of algae was transferred into a chromatographic vial, then 25 μL of the internal standard tripentadecanoin (C15:0; 1000 μg/mL), 200 μL of dichloromethane:methanol (2:1 *v*/*v*), and 300 μL of 0.6 M HCl were added to the sample. The samples were sealed, vigorously mixed, and heated to 85 ± 3 °C for 1 h in a laboratory oven (S-40, Alpina, Konin, Poland). Afterward, the samples were cooled to room temperature, and 1 mL of petroleum ether was added. The components in the vials were mixed again for 1 min and left for 1 h to allow for phase separation. Then, 100 μL from the upper phase was transferred to a 2 mL chromatographic vial, and 400 μL of petroleum ether was added.

The analysis was performed on a 7890A gas chromatograph (Agilent Technologies, Palo Alto, CA, USA) equipped with a mass detector, model 7000 (GC-MS/MS QqQ). The mixtures were separated on the HP-5 MS Ultra Inert Capillary Column (30 m × 0.25 mm ID × 0.25-μm). The mass spectrometer was used in electron impact (EI) ionization mode. Ions were monitored in full-scan mode in the range of 50 *m*/*z* to 400 *m*/*z*. The analyses were conducted in a programmed mode with a temperature gradient of 40–260 °C. For the identification and quantification of fatty acids, an external standard was used (Supelco 37 component FAME MIX CRM47885, Merck, KGaA, Darmstadt, Germany).

### 4.3. Chlorophylls and Carotenoids

The content of pigments (chlorophylls and carotenoids) was assayed in dry biomass (50 mg) as described in [75]. The pigments were extracted with ice-cold acetone containing 0.1% butylated hydroxytoluene (BHT; Sigma-Aldrich, Poznan, Poland). Ultrasonic-assisted extraction was performed using a Sonic-14 ultrasonic bath (Polsonic, Warsaw, Poland) operated at a frequency of 40 kHz and an ultrasonic input power of 2 × 400 W. The absorption was measured using a Cary 50 Bio UV-Vis spectrophotometer (Varian, Belrose, NSW, Australia), at λ = 644.8 nm, 661.6 nm, and 470 nm against 100% acetone as a blank. The following equations were used to ascertain the concentration of chlorophyll *a*, chlorophyll *b*, and carotenoids. The pigment content was expressed as micrograms per gram of dry weight (D.W.) ± SD. The following calculations were used to determine the levels of chlorophylls and carotenoids:

C_a_ = 11.24A_661.6_ − 2.04A_644.8_

C_b_ = 20.13A_644.8_ − 4.19A_661.6_

C_x+c_ = (1000A_470_ − 1.90C_a_ − 63.14C_b_)/214

C_a_—Chlorophyll *a*; C_b_—Chlorophyll *b*; C_x+c_—Carotenoids

### 4.4. Total Phenols

Phenolic compounds were extracted from 50 mg of dried algal biomass with 5 mL of 80% MeOH for 30 min using an ultrasonic bath (Sonic-14, Polsonic, Poland). The extract was centrifuged at 5000× *g*, and the supernatant was collected. The extraction was then repeated. The combined supernatants were evaporated under vacuum at 40 °C (Hei-VAP Precision, Heidolph Instruments GmbH & Co. KG, Schwabach, Germany). The residue was suspended in water and applied onto a Chromabond C18ec column, which was activated with methanol and equilibrated with water (Macherey-Nagel GmbH & Co. KG, Düren, Germany). The separation was carried out using a SPE Vacuum Manifold system (Sigma-Aldrich, Poznan, Poland). Subsequently, the column was washed with H_2_O, and the phenolic compounds were eluted with methanol.

Total phenolic compounds were determined spectrophotometrically using the Folin–Ciocalteu reagent (Sigma-Aldrich, Poznan, Poland). Briefly, 0.075 mL of the sample was mixed with 0.975 mL of H_2_O, and then 0.075 mL of Folin–Ciocalteu reagent (diluted 1:1 with water) was added. After 3 min of incubation in the dark at room temperature, 0.125 mL of 20% Na_2_CO_3_ (Sigma-Aldrich, Poznan, Poland) was added and mixed. The absorbance of the blue complex was measured at 725 nm using an Epoch microplate spectrophotometer (BioTek Instruments Inc., Winooski, VT, USA). Total phenolic content was expressed as gallic acid equivalents based on the calibration curve.

### 4.5. L-Phenylalanine Ammonia-Lyase (PAL) Activity

The activity of L-phenylalanine ammonia-lyase (PAL) was determined in accordance with [26]. Prior to the enzymatic analysis, the samples were treated with 6 mL of ice-cold acetone. Subsequently, 200 mg of the acetone dry powder was homogenized with 5 mL of ice-cold borate buffer (0.1 M, pH 8.8). The reaction mixture consisted of 0.25 mL of extract, 0.5 mL of substrate (L-phenylalanine, 0.03 M, Sigma-Aldrich, Poznan, Poland), 0.5 mL of borate buffer (0.1 M, pH 8.8), and 0.25 mL of distilled water. The reaction mixture was incubated for 45 min at 30 °C, then 0.5 mL of TCA (Sigma-Aldrich, Poznan, Poland) was added, and the mixture was centrifuged for 20 min at 6000× *g*. The absorbance of the produced trans-cinnamic acid was measured at 290 nm using a Cary 50 Bio UV-Vis spectrophotometer (Varian, Belrose, NSW, Australia). PAL activity was expressed as nanomoles of produced trans-cinnamic acid per 1 g of D.W. algal cells per minute, based on the molar coefficient (ε = 9.3 mM^−1^ × cm^−1^) obtained for trans-cinnamic acid dissolved in borate buffer (0.1 M, pH 8.8).

### 4.6. Statistical Analysis

All chemical analyses were conducted in triplicate. Microalgal size, cell number, content of fatty acids, total phenols, and enzyme activity were expressed as the mean ± standard deviation. Statistical analysis was performed using the Statistica, ver. 13.3 Software (TIBCO Software, San Ramon, CA, USA). Data were subjected to ANOVA, with salicylate type and concentration as fixed effects. The significance of the differences between mean values was calculated using Tukey’s multiple comparison post hoc test, with a *p*-value of < 0.05 considered statistically significant.

## 5. Conclusions

This study demonstrated that salicylic acid (SA) and methyl salicylate (MS) act as effective elicitors of secondary metabolism in *Planktochlorella nurekis*, with responses that depend on the applied concentration and chemical form. We demonstrated that, although MS is considered a volatile, inactive form of SA, it elicited *P. nurekis* secondary metabolism most intensely, resulting in the highest accumulation of carotenoids, chlorophyll *b*, and phenolic compounds, along with the strongest L-phenylalanine ammonia-lyase activity. The rise in PAL activity suggests that the phenylpropanoid pathway in *P. nurekis* can be induced and proceed via this enzyme, whose occurrence in microalgae remains poorly documented. Furthermore, both salicylates shifted the fatty acid composition towards monounsaturated forms, which offer potent nutritional benefits. Future developments could focus on establishing the yields of individual compounds and discovering the transcriptional bases of microalgal responses. Moreover, optimizing salicylate application may prove valuable for the industry, as our results indicate that growth stimulation and metabolic enrichment respond to different forms and concentrations of salicylates; therefore, these elicitors can be deployed sequentially in a two-stage cultivation, first by promoting biomass accumulation and then by enriching the composition of the microalgae.

## Figures and Tables

**Table 1:** Fatty acid profiles within *P. nurekis* cells under different concentrations of SA and MS treatments. Values are expressed as the average content (in %) ± SD of three replicates ^1^.

Identified Fatty Acid	Control	SA	SA	SA	MS	MS	MS
		0.1 µM	1 µM	10 µM	0.1 µM	1 µM	10 µM
Myristic (C14:0)	2.11 ± 0.12 ^g^	1.94 ± 0.10 ^f^	1.60 ± 0.09 ^ef^ **	1.57 ± 0.09 ^f^ **	2.06 ± 0.11 ^f^	1.55 ± 0.08 ^f^ **	1.91 ± 0.10 ^g^
Palmitic (C16:0)	12.25 ± 0.64 ^d^	13.69 ± 0.72 ^c^	10.15 ± 0.53 ^c^ **	9.78 ± 0.52 ^d^ **	11.53 ± 0.60 ^c^	11.13 ± 0.59 ^c^	11.19 ± 0.60 ^d^
Margaric (C17:0)	0.51 ± 0.02 ^h^	0.51 ± 0.02 ^e^	1.16 ± 0.01 ^ef^ **	0.72 ± 0.04 ^f^ **	0.60 ± 0.05 ^fg^ **	0.51 ± 0.01 ^f^	0.61 ± 0.01 ^gh^ **
Stearic (C18:0)	6.10 ± 0.31 ^e^	4.77 ± 0.21 ^g^ **	7.81 ± 0.14 ^d^ **	4.99 ± 0.25 ^e^ **	5.69 ± 0.29 ^d^	6.71 ± 0.31 ^d^	6.08 ± 0.15 ^e^
Arachidic (C20:0)	0.24 ± 0.01 ^h^	0.22 ± 0.01 ^g^	0.29 ± 0.02 ^f^ **	0.22 ± 0.01 ^f^	0.24 ± 0.01 ^g^	0.25 ± 0.02 ^f^	0.27 ± 0.01 ^gh^
Behenic (C22:0)	0.18 ± 0.01 ^h^	0.18 ± 0.01 ^g^	0.18 ± 0.01 ^f^	0.14 ± 0.01 ^f^ **	0.15 ± 0.01 ^g^ *	0.16 ± 0.01 ^f^	0.18 ± 0.01 ^h^
Tricosylic (C23:0)	0.09 ± 0.02 ^h^	0.09 ± 0.01 ^g^	0.09 ± 0.03 ^f^	0.07 ± 0.02 ^f^ **	0.07 ± 0.10 ^g^ *	0.08 ± 0.02 ^f^ *	0.08 ± 0.02 ^h^
Lignoceric (C24:0)	0.29 ± 0.02 ^h^	0.21 ± 0.01 ^g^ **	0.18 ± 0.01 ^f^ **	0.17 ± 0.01 ^f^ **	0.19 ± 0.01 ^g^ **	0.24 ± 0.01 ^f^ **	0.28 ± 0.02 ^gh^
Myristoleic (C14:1n−5)	0.05 ± 0.01 ^h^	0.06 ± 0.01 ^g^	0.04 ± 0.00 ^f^	0.03 ± 0.01 ^f^	0.03 ± 0.01 ^g^ *	0.04 ± 0.01 ^f^	0.04 ± 0.01 ^h^
Pentadecenoic (C15:1)	0.04 ± 0.00 ^h^	0.04 ± 0.01 ^e^	0.03 ± 0.01 ^f^	0.03 ± 0.01 ^f^	0.03 ± 0.01 ^g^	0.03 ± 0.01 ^f^	0.03 ± 0.01 ^h^
Palmitoleic (C16:1n−7)	3.77 ± 0.22 ^f^	4.75 ± 0.20 ^g^ **	2.12 ± 0.10 ^e^ **	3.58 ± 0.18 ^e^	3.68 ± 0.22 ^e^	3.00 ± 0.14 ^e^ **	3.46 ± 0.19 ^f^
Heptadecenoic (C17:1)	0.16 ± 0.01 ^h^	0.30 ± 0.01 ^b^ **	0.29 ± 0.01 ^f^ **	0.27 ± 0.03 ^f^ **	0.16 ± 0.02 ^g^	0.18 ± 0.01 ^f^ *	0.17 ± 0.01 ^h^
Oleic (C18:1n−9, cis)	24.79 ± 1.08 ^b^	29.02 ± 1.61 ^fg^	31.45 ± 1.51 ^a^ **	35.94 ± 1.58 ^a^ **	30.13 ± 1.59 ^a^ *	30.18 ± 1.66 ^a^ *	35.99 ± 1.90 ^a^ **
Gondoic (C20:1n−9)	0.19 ± 0.02 ^h^	0.21 ± 0.01 ^g^ *	0.21 ± 0.01 ^f^ *	0.22 ± 0.01 ^f^ **	0.20± 0.01 ^g^	0.17 ± 0.01 ^f^	0.21 ± 0.01 ^h^
Nervonic (C24:1n−9)	0.04 ± 0.00 ^h^	0.04 ± 0.01 ^g^	0.03 ± 0.01 ^f^	0.03 ± 0.01 ^f^	0.04 ± 0.02 ^g^	0.04 ± 0.01 ^f^	0.04 ± 0.02 ^h^
Linoleic (C18:2n−6, cis)	31.86 ± 1.56 ^a^	34.78 ± 1.50 ^a^	32.08 ± 1.43 ^a^	29.36 ± 1.48 ^b^	30.59 ± 1.56 ^a^	29.06 ± 1.22 ^a^	25.77 ± 1.36 ^b^ **
Eicosatrienoic (C20:3n−3)	0.07 ± 0.02 ^h^	0.08 ± 0.02 ^g^	0.06 ± 0.01 ^f^ *	0.06 ± 0.01 ^f^ **	0.06 ± 0.01 ^g^ **	0.06 ± 0.00 ^f^ **	0.06 ± 0.01 ^h^
Arachidonic (C20:4n−6)	0.10 ± 0.00 ^h^	0.10 ± 0.01 ^g^	0.08 ± 0.02 ^f^ **	0.07 ± 0.02 ^f^ **	0.00 ± 0.00 ^g^ **	0.00 ± 0.00 ^g^ **	0.09 ± 0.02 ^h^
Eicosapentaenoic (C20:5n−3)	0.06 ± 0.01 ^h^	0.06 ± 0.01 ^g^	0.04 ± 0.01 ^f^	0.03 ± 0.01 ^f^ **	0.04 ± 0.01 ^g^ **	0.05 ± 0.01 ^f^	0.04 ± 0.01 ^h^ *
Docosahexaenoic (C22:6n−3)	0.04 ± 0.01 ^h^	0.06 ± 0.00 ^g^ **	0.05 ± 0.01 ^f^	0.04 ± 0.01 ^f^	0.04 ± 0.00 ^g^	0.06 ± 0.02 ^f^ **	0.08 ± 0.01 ^h^ **
γ-Linolenic (C18:3n−6)	0.07 ± 0.02 ^h^	0.09 ± 0.00 ^g^ **	0.06 ± 0.01 ^f^	0.07 ± 0.02 ^f^	0.05 ± 0.01 ^g^	0.06 ± 0.01 ^f^	0.14 ± 0.01 ^h^ **
α-Linolenic (C18:3n−3)	17.00 ± 0.73 ^c^	8.80 ± 0.37 ^d^ **	12.01 ± 0.64 ^b^ **	12.63 ± 0.69 ^c^ **	14.42 ± 0.68 ^b^ **	16.39 ± 0.72 ^b^	13.27 ± 0.64 ^c^ **
SAFAs ^2^	21.75 ± 0.19	21.58 ± 0.22	21.53 ± 0.86	17.64 ± 0.84 **	20.52 ± 0.44	20.67 ± 0.24	20.59 ± 0.90
MUFAs ^2^	29.11 ± 0.99	34.54 ± 1.34	34.11 ± 1.71 *	40.22 ± 1.54 **	34.31 ± 1.78 *	33.62 ± 1.79 *	39.92 ± 1.64 **
PUFAs ^2^	49.14 ± 0.80	43.88 ± 1.12 **	44.36 ± 0.85 **	42.15 ± 0.71 **	45.18 ± 2.23 *	45.72 ± 2.02	39.49 ± 0.74 **

^1^ The symbol n in abbreviated fatty acid names describes the location of the last double bond in the chain, and thus the type of omega acid. Means within the columns followed by lowercase letters indicate statistically significant differences among fatty acids according to Tukey’s test (*p* < 0.05). Asterisks indicate significant differences between treatments and the control group evaluated by Student’s *t*-test (* *p* < 0.05, ** *p* < 0.01). ^2^ SAFAs (Saturated Fatty Acids); MUFAs (Monounsaturated Fatty Acids); PUFAs (Polyunsaturated Fatty Acids).

## Data Availability

The original data presented in this study are included in the article. Further inquiries can be directed to the corresponding author.

## Abbreviations

The following abbreviations are used in this manuscript:

6-BA	6-Benzylaminopurine
BHT	Butylated Hydroxytoluene
CCD	Charge-Coupled Device
CHI	Chalcone Isomerase
CHS	Chalcone Synthase
D.W.	Dry Weight
EBR	Epibrassinolide
EDTA	Ethylenediaminetetraacetic Acid
EI	Electron Impact Ionisation
FAME	Fatty Acid Methyl Ester
FAS	Fatty Acid Synthase
FHT	Flavanone 3β-Hydroxylase
GC-MS	Gas Chromatography–Mass Spectrometry
GGPP	Geranylgeranyl Pyrophosphate
IPP	Isopentenyl Pyrophosphate
LCYB	Lycopene Β-Cyclase
LED	Light-Emitting Diode
LHC	Light-Harvesting Complex
MAPK	Mitogen-Activated Protein Kinase
MEP	2-C-Methyl-D-Erythritol 4-Phosphate
MS	Methyl Salicylate
MUFA	Monounsaturated Fatty Acid
NAA	1-Naphthaleneacetic Acid
NF-κB	Nuclear Factor Kappa B
NPR1	Nonexpressor Of Pathogenesis-Related Genes 1
OD	Optical Density
PAL	L-Phenylalanine Ammonia-Lyase
PDS	Phytoene Desaturase
PSY	Phytoene Synthase
PTD9	Δ^9^-desaturase
PUFA	Polyunsaturated Fatty Acid
ROS	Reactive Oxygen Species
SA	Salicylic Acid
SABP	Salicylic Acid-Binding Protein
SAFA	Saturated Fatty Acid
SAR	Systemic Acquired Resistance
SD	Standard Deviation
SPE	Solid Phase Extraction
TAG	Triacylglycerol
TAL	L-Tyrosine Ammonia-Lyase
TCA	Trichloroacetic Acid
